# Use of a Molecular Diagnostic Test in AFB Smear Positive Tuberculosis Suspects Greatly Reduces Time to Detection of Multidrug Resistant Tuberculosis

**DOI:** 10.1371/journal.pone.0031563

**Published:** 2012-02-09

**Authors:** Nestani Tukvadze, Russell R. Kempker, Iagor Kalandadze, Ekaterina Kurbatova, Michael K. Leonard, Rusudan Apsindzelashvili, Nino Bablishvili, Maia Kipiani, Henry M. Blumberg

**Affiliations:** 1 National Center for Tuberculosis and Lung Diseases, Tbilisi, Georgia; 2 Emory University School of Medicine, Division of Infectious Diseases, Atlanta, Georgia, United States of America; University of Cape Town, South Africa

## Abstract

**Background:**

The WHO has recommended the implementation of rapid diagnostic tests to detect and help combat M/XDR tuberculosis (TB). There are limited data on the performance and impact of these tests in field settings.

**Methods:**

The performance of the commercially available Genotype MTBDR*plus* molecular assay was compared to conventional methods including AFB smear, culture and drug susceptibility testing (DST) using both an absolute concentration method on Löwenstein-Jensen media and broth-based method using the MGIT 960 system. Sputum specimens were obtained from TB suspects in the country of Georgia who received care through the National TB Program.

**Results:**

Among 500 AFB smear-positive sputum specimens, 458 (91.6%) had both a positive sputum culture for *Mycobacterium tuberculosis* and a valid MTBDR*plus* assay result. The MTBDR*plus* assay detected isoniazid (INH) resistance directly from the sputum specimen in 159 (89.8%) of 177 specimens and MDR-TB in 109 (95.6%) of 114 specimens compared to conventional methods. There was high agreement between the MTBDR*plus* assay and conventional DST results in detecting MDR-TB (kappa = 0.95, p<0.01). The most prevalent INH resistance mutation was S315T (78%) in the *katG* codon and the most common rifampicin resistance mutation was S531L (68%) in the *rpoB* codon. Among 13 specimens from TB suspects with negative sputum cultures, 7 had a positive MTBDR*plus* assay (3 with MDR-TB). The time to detection of MDR-TB was significantly less using the MTBDR*plus* assay (4.2 days) compared to the use of standard phenotypic tests (67.3 days with solid media and 21.6 days with broth-based media).

**Conclusions:**

Compared to conventional methods, the MTBDR*plus* assay had high accuracy and significantly reduced time to detection of MDR-TB in an area with high MDR-TB prevalence. The use of rapid molecular diagnostic tests for TB and drug resistance should increase the proportion of patients promptly placed on appropriate therapy.

## Introduction

The global emergence of multidrug-resistant (MDR) tuberculosis (resistance to isoniazid [INH] and rifampicin [RIF]) is an alarming issue in international tuberculosis (TB) control and presents an enormous challenge not yet sufficiently addressed [1]. The latest global surveillance data indicate the highest level of drug-resistance ever recorded with an estimated 440,000 MDR-TB cases worldwide resulting in 150,000 deaths in 2009 [2]. MDR-TB has proven difficult to treat due to costly, complex, and less effective treatment regimens and is associated with significantly worse outcomes as compared to drug susceptible disease [3]. Of particular concern is that only an estimated 7% of all MDR-TB cases are detected [2]. Conventional AFB culture and drug susceptibility testing (DST) requires significant laboratory infrastructure and has a slow turnaround time which can result in delayed initiation of proper therapy and increasing risk of disease transmission and amplification of drug resistance due to initiation of inadequate treatment regimens [4]. In response to the growing problem of MDR-TB, the STOP TB strategy has made universal access to diagnosis and treatment of MDR-TB a priority with a focus on rapid MDR-TB detection [5].

Responding to the urgent need for rapid diagnostic tests, several molecular based methods have been developed in the last few years including the commercially available line probe assay, the Genotype MTBDR*plus* assay [Hain Lifescience] [6]. The Genotype MTBDR*plus* assay uses DNA amplification followed by reverse hybridization to detect the presence of *M. tuberculosis* DNA and the most common genetic mutations conferring resistance to RIF (in *rpoB* gene) and INH (in *katG* and *inhA* genes). Trained personnel can perform the test within 8 hours. A recent meta-analysis found the Genotype MTBDR*plus* assay performed well, as compared to conventional DST [7]. Based on available data and expert opinion the WHO has approved the use of line probe assays (LPA) for rapid MDR-TB screening; specifically recommending testing in patients with acid-fast bacilli (AFB) positive smears and the use of commercial LPAs [8]. While tests such as the Genotype MTBDR*plus* assay offer great promise to improving MDR-TB detection and care, the urgent need for operational research evaluating test performance in a real-world setting has been highlighted [9].

Georgia (a former Soviet republic) is one of twenty-seven high burden MDR-TB countries as designated by the WHO [2]. In 2009, the TB incidence rate in Georgia was 100 per 100,000. The prevalence of MDR in Georgia in 2009 was 10.3% in newly diagnosed patients and 31.1% in previously treated patients [10]. With the support of the Global Fund and the Green Light Committee (GLC), Georgia has became one of the first low and middle income countries to achieve universal access to diagnosis and treatment of MDR-TB beginning in 2008. The primary objective of our study was to assess the performance, impact, and time to detection of drug resistant TB of a rapid molecular diagnostic test compared to conventional culture and DST methods when implemented into the normal workflow of a high volume National TB Reference Laboratory (NRL) which provides laboratory support for the Georgian National TB Program (NTP).

## Methods

### Study Setting and Population

The study took place at the NRL of the Georgian NTP in Tbilisi, Georgia. The NRL processes specimens for the entire country of Georgia. Approximately 15,000 sputum specimens were processed at the NRL in 2010.

Between June and October 2009, all AFB smear positive sputum specimens obtained from TB suspects without previous history of TB from throughout Georgia were consecutively enrolled into the study. Subsequently, from February through July 2010, all TB cases with AFB smear positive sputum specimens (regardless of prior treatment status) from Tbilisi, Georgia were consecutively enrolled into the study. All testing was performed on routine clinical sputum specimens.

### Ethics Statement

The study was conducted according to the principles of the Declaration of Helsinki. The Georgian NTP and Emory University Institutional Review Boards approved the study and granted a waiver of informed consent for the study. All samples were de-identified of personal identifiers for data entry and data analysis.

### Culture and Drug Susceptibility Testing (DST)

Three sputum specimens were obtained from each patient at NTP sputum microscopy centers throughout the country. Direct smears with Ziehl-Neelsen staining were examined by light microscopy at local microscopy centers. One AFB smear positive sputum sample was sent to NRL in Tbilisi where it was processed using standard methodologies (decontaminated in a BSL3 area with N-acetyl-L-cysteine-sodium hydroxide, centrifuged, and the sediment was then suspended in 1.5 ml of phosphate buffer) [11]. The processed specimen was inoculated on to both Löwenstein-Jensen (LJ) based solid medium and the BACTEC MGIT 960 broth culture system. The duration of incubation for LJ solid culture was 60 days and for MGIT broth culture 42 days. Positive cultures by either method were confirmed to be *Mycobacterium tuberculosis* complex (MTBC) using the MTBDR*plus* assay along with colony morphology [6]. DST for INH and RIF was performed using either the absolute concentration method on LJ medium (INH 0.2 µg/ml, RIF 40 µg/ml) or in 7H9 broth with the BACTECT MGIT 960 system (INH 0.1 µg/ml, RIF 1 µg/ml) [12]. DST to second-line drugs (SLDs) was performed using the proportion method on LJ medium with the following drug concentrations: ethionamide-40.0 µg/ml; ofloxacin-2.0 µg/ml; para-aminosalicylic acid-0.5 µg/ml, capreomycin-40.0 µg/ml and KM-30.0 µg/ml [13]. The NRL has undergone external quality assessment by the Antwerp WHO Supranational TB Reference Laboratory annually since 2005. The last round of quality control for first-line drugs was performed in 2009 with 97% accuracy for INH and 100% for RIF.

### Genotype MTBDRplus Assay

The MTBDR*plus* assay was performed directly on sputum samples and according to the manufacturer's instructions [6]. A portion of the same sputum specimen was used for both molecular testing and culture at the NRL. A 500-µl portion of decontaminated samples was used for DNA isolation; subsequent amplification and hybridization was based on manufacturers recommendations [6]. Each step was carried out in a separate room with unidirectional workflow between rooms. After hybridization, test strips were allowed to dry before attached to paper. Each strip consists of 27 reaction zones (bands) including controls that were interpreted according to manufacturers instructions to determine test validity, MTBC identification, and resistance to INH and RIF. An internal quality control program with positive and negative controls was implemented during the study. The MTBDR*plus* assay was performed two to three times per week with between 2–8 samples used per run.

### Definitions

INH mono-resistance was defined as *M. tuberculosis* resistance to INH without resistance to RIF. RIF mono-resistance was defined as resistance to RIF without resistance to INH. MDR-TB was defined as resistance to both INH and RIF. New cases were defined as patients who had received ≤30 days of TB treatment and retreatment cases as all patients with a prior history of receiving TB treatment for >30 days. A completely interpretable MTBDRplus result was defined as a test strip with all control markers positive.

### Data Analysis

All data were entered into an online REDCap database [14] and analyzed using SAS 9.3 (SAS Institute Inc., Cary, NC). The sensitivity, specificity, positive predictive value (PPV), and negative predictive value (NPV) of the MTBDR*plus* assay in detecting resistance to INH, RIF, and MDR were calculated using conventional culture and DST results as the reference standard. Turnaround time was calculated as time between the date of sputum collection and date of culture, DST, and MTBDR*plus* test results. The degree of agreement between test results were assessed using the kappa (κ) statistic with a value of κ = 1 denoting perfect concordance, and k = 0 denotes agreement by chance alone. A p-value of <0.05 was considered statistically significant.

## Results

**Figure 1:**
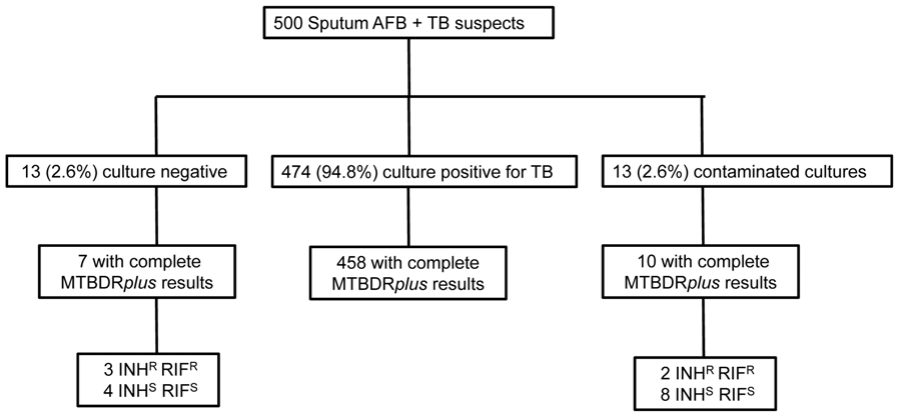
Sputum culture results for all AFB smear positive tuberculosis suspects and corresponding complete MTBDRplus assay results.

**Figure 2:**
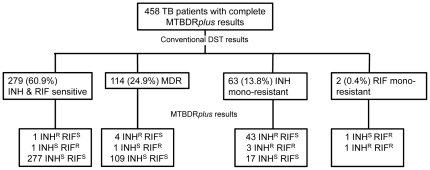
Distribution of MTBDR*plus* assay results according to phenotypic drug resistance patterns using conventional drug susceptibility testing for specimens with both valid culture and MTBDRplus assay results.

A total of 500 patients with suspected pulmonary TB who had a smear positive AFB sputum specimen were enrolled into the study. Among these 500 patients, 379 (76%) had no prior history of TB and 121 (24%) were retreatment TB cases. Overall, 474 (95%) samples were culture positive by either conventional method (92% had positive liquid culture, and 79% positive solid culture), 2.6% were culture negative, and 2.6% had contaminated cultures (Figure 1). All culture negative cases had 1+ AFB smear positivity. DST of M. tuberculosis was performed in liquid media in 325 (69%) or solid media in 149 (31%). Conventional DST revealed 114 (25%) *M. tuberculosis* (MTB) isolates had multidrug resistance, 63 (14%) were isoniazid mono-resistance, and 2 (0.4%) isolates were rifampin mono-resistant (Figure 2). There was a much higher rate of MDR TB among patients with a prior history of TB treatment as compared to persons never treated for TB (54% vs. 16%, p<0.05). Second line drug susceptibility testing of MDR isolates revealed 11(10% of MDR cases and 2% of all culture positive cases) had XDR. Of the 11 XDR cases 4 of 47 were new TB cases and 7 of 51 were retreatment TB cases.

### MTBDR*plus* Assay Performance

The MTBDR*plus* assay identified the presence of *M. tuberculosis* in 485 (97%) of 500 sputum samples and had completely interpretable results in 475 (95%). Overall, there was no significant difference in the proportion of interpretable results between conventional methods and the MTBDR*plus* assay (97% vs. 97%, p = 0.90). Among 474 sputum samples which subsequently yielded a positive culture for *M. tuberculosis*, 458 (97%) had a completely interpretable MTBDR*plus* assay. Of the 16 results with positive culture and incomplete MTBDR*plus* assay results, the MTBDR*plus* assay identified MTBC in 10 but did not have complete amplification of the RIF and/or INH bands, and in 6 samples had control amplification but did not identify the presence of *M. tuberculosis* DNA, indicating the presence of a non-tuberculous *Mycobacterium*. Figure 2 shows MTBDR*plus* results for four different categories of results of drug resistance testing as detected by conventional DST. Performance parameters of the MTBDR*plus* assay as compared to conventional DST for detection of RIF, INH, and MDR are displayed in Table 1. The overall sensitivity, specificity, positive predictive value, and negative predictive value of the MTBDR*plus* assay in the detection of RIF resistance, INH resistance, and MDR were high (Table 1). The sensitivity of the MTBDR*plus* assay in detection of INH resistance among isolates with INH mono-resistance was less than the detection of INH resistance in MDR-TB isolates (73% vs. 99%, p<0.05). There was high agreement between the MTBDR*plus* assay and conventional DST in detection of RIF resistance (k = 0.94; 95% CI 0.91–0.98), INH resistance (k = 0.91; 95% CI 0.87–0.95), and MDR (k = 0.95; 95% CI 0.92–0.99). The performance of the MTBDR*plus* assay was similar in cases with or without prior treatment for TB except decreased sensitivity of INH resistance detection in persons without prior treatment for TB as compared to persons with prior TB treatment (86.0% vs. 95.7%, p<0.05).

**Table 1:** Performance parameters of MTBDR*plus* in detecting INH R∧, RIF R∧, and MDR∧ compared to conventional DST (reference standard)#.

	Isoniazid	Rifampicin	Multidrug Resistance
Sensitivity	89.8 (84.4–93.9)	96.6 (91.4–99.1)	95.6 (90.1–98.6)
Specificity	99.3 (97.5–99.9)	98.8 (97.0–99.7)	98.5 (96.6–99.5)
PPV*	98.8 (95.6–99.9)	96.6 (91.4–99.1)	95.6 (90.1–98.6)
NPV*	93.3 (90.6–96.4)	98.8 (97.0–99.7)	98.5 (96.6–99.5)

# Values are percentages with 95% confidence interval in parentheses.

*PPV = positive predictive value, NPV = negative predictive value.

∧ INH R = isoniazid resistance, RIF R = rifampin resistance, MDR = multidrug resistance.

The MTBDR*plus* assay gave interpretable results for the majority (17 of 26, 65%) of specimens with negative or contaminated sputum cultures (Figure 1). In 13 sputum samples from patients suspected of having pulmonary TB but in whom the sputum culture had no growth, the MTBDR*plus* assay identified *M. tuberculosis* in 7 (54%) specimens including 3 specimens in which multidrug resistance was identified by the MTBDR*plus* assay. In the 13 specimens with contaminated cultures, the MTBDRplus assay identified MTBC complex in 10 (77%) specimens of which 2 were MDR-TB.

### Time to Results

Time to detection of *M. tuberculosis* and drug resistance were significantly shorter for the MTBDR*plus* assay compared to conventional methods using solid and liquid culture and DST (Table 2). On average, a MTBDR*plus* test result for the detection of *M. tuberculosis* was available by 4.2 days (1SD+/−1.8 days) while positive solid culture and liquid culture results were not available until 34.1 (1SD+/−11.3 days) and 8.9 days (1SD+/−3.9 days), respectively. In regards to drug resistance, the MTBDR*plus* result for detection for INH and/or RIF resistance was available by 4.2 days (1SD+/−1.8 days) as compared to solid and liquid media DST results which were not available until 67.5 (1SD+/−15.0) and 21.6 days (1SD+/−9.3 days), respectively. The time to detection of drug resistance was similar for MDR or mono-resistant specimens.

**Table 2:** Average time to results in days for detection of TB and associated drug resistance (N = 458)*.

	Positive Solid Culture Result	Solid Media DST∧	Positive Liquid Culture Result	Liquid Media DST∧	MTBDR*plus* assay
**All Cases**	34.1 (11.3)	67.5 (15.0)	8.9 (3.9)	21.6 (9.3)	4.2 (1.8)
**MDR TB**	36.9 (13.4)	70.4 (19.2)	8.9 (4.9)	21.1 (8.1)	3.7 (1.7)

*Values are average number of days with one standard deviation in parentheses.

∧ DST = drug susceptibility testing.

### Genetic Mutations

The distribution of genetic mutations of drug-resistant *M. tuberculosis* isolates with an interpretable MTBDR*plus* assay (n = 179) is shown in Table 3. The most common resistance mutation for INH was S315T (78%) in the *katG* codon followed by C15T (28%) in the *inhA* codon. Additionally, a high percentage of isolates (72%) had no binding to the *katG* WT probe. Overall, 18 (10%) of 177 INH resistant isolates had a genetic abnormality isolated to the *InhA* codon; but this mutation was significantly more common in INH mono-resistant isolates compared to strains with MDR resistance (21% vs. 4%, p<0.05). In comparing MDR to INH mono-resistant isolates, MDR isolates had an increased frequency of the S315T mutation (92% vs. 52%, p<0.05) and less binding to the *katG* WT probe (10% vs. 54%, p<0.05). Isolates from persons with prior TB treatment were more likely to have either a genetic mutation in the *katG* codon or lack of binding to *katG* WT probe (95.5% vs. 83.4%, p<0.05) and an increased likelihood of having a genetic mutation or lack of binding to WT gene region in both *katG* and *inhA* codons (23.9%. vs. 17.2%, p = 0.29). In RIF resistant isolates, the most common genetic abnormality was the lack of binding to the WT8 probe in 80%, followed by the S531L mutation in 68%. Of the five isolates with RIF resistance by the MTBDR*plus* assay but RIF S by conventional DST all five were RIF resistance due to the lack of binding to one or more WT bands with no mutation bands present.

**Table 3:** Pattern of genetic mutations in phenotypic drug-resistant *Mycobacterium tuberculosis* isolates using the Genotype MTBDRplus assay.

Gene	Band	Gene Region of Mutation	IHN Mono Resistance* ^,^ ∧n = 63	RIF Mono Resistance* ^,^ ∧n = 2	MDR* ^,^ ∧n = 114
*katG*	WT	315	34 (54)	-	15 (10)
	MUT1	S315T1	33 (52)	-	105 (92)
	MUT2	S315T2	0	-	3 (3)
*inhA*	WT1	−15/−16	47 (75)	-	85 (75)
	WT2	−8	62 (98)	-	114 (100)
	MUT1	C15T	17 (27)	-	33 (30)
	MUT2	A16G	0	-	1 (1)
	MUT3A	T8C	0	-	0
	MUT3B	T8A	0	-	0
*rpoB*	WT1	506–509	-	2 (100)	114 (100)
	WT2	510–513	-	2 (100)	114 (100)
	WT3	513–517	-	2 (100)	106 (93)
	WT4	516–519	-	2 (100)	106 (93)
	WT5	518–522	-	2 (100)	114 (100)
	WT6	521–525	-	2 (100)	112 (98)
	WT7	526–529	-	2 (100)	108 (95)
	WT8	530–533	-	0	22 (19)
	MUT1	D516V	-	0	7 (6)
	MUT2A	H526Y	-	0	2 (2)
	MUT2B	H526B	-	0	2 (2)
	MUT3	S531L	-	1 (50)	78 (68)

*Definitions of abbreviations: INH = isoniazid; RIF = rifampicin; MDR = multidrug-resistant.

∧ Values are numbers, with percentages in parentheses.

### Predicting Ethionamide Resistance

Among 109 MDR-TB isolates with DST performed for second line drugs, 102 (94%) were found to be resistant to ethionamide. While the sensitivity and negative predictive value of any abnormality in the i*nhA* gene in detecting ethionamide resistance were low at 29% and 9% respectively, the specificity and positive predictive value were both 100%.

## Discussion

In a high-burdened MDR-TB country, the MTBDR*plus* assay performed extremely well in the detection of *M. tuberculosis* complex and MDR-TB as compared to conventional culture and DST. This rapid molecular diagnostic test can be performed directly on sputum samples from patients with suspected pulmonary TB and demonstrated a high accuracy in our study detecting *M. tuberculosis* and for detecting INH and RIF resistance compared to conventional culture and DST methodologies. In addition, the MTBDR*plus* detected drug resistance much more quickly than conventional methods (3.7 days with MTBDR*plus* assay vs. 21.1 days with liquid media DST vs. 70.4 with solid media DST); a finding which has great implications in improving the clinical care of MDR-TB patients. The results demonstrate line probe assays can be successfully implemented into the routine workflow of a high volume national reference laboratory.

The high sensitivity (95.6%), specificity (98.5%), PPV (95.6%), and NPV (98.5%) in the detection of MDR-TB in our study correlate well with findings from a previous report evaluating the MTBDR*plus* assay in South Africa [15]. While other studies assessing the MTBDR*plus* assay found high test accuracy, most were performed on stored samples and in a purely research setting [16], [17], [18], thus limiting the generalizability of the results for routine clinical practice. Additionally, we found that the MTBDR*plus* gave valid results in majority of cases where there was culture contamination (77%) or no culture growth (54%) demonstrating the MTBDR*plus* test may offer superior performance to conventional methods. Five MDR-TB cases were detected by the MTBDR*plus* test only; otherwise these cases would have gone undetected due to no culture growth (n = 3) or contamination (n = 2). Similarly, there were five cases found to MDR by conventional DST but only INH (n = 4) or RIF (n = 1) mono resistance by the MTBDR*plus* assay. It has been speculated that samples with a valid MTBDR*plus* test result but no growth on culture may be the result of excess decontamination, which can kill a high percentage of mycobacteria in a specimen [19].

While the sensitivity of the molecular diagnostic test for detection of INH resistance in our study was slightly lower than that for RIF and MDR detection (compared to conventional methods), it was in line with prior studies [7]. The slightly lower sensitivity of the MTBDR*plus* test for INH compared to conventional methods is likely due to genetic mutations conferring INH resistance that are located outside the *katG* and *inhA* genes [20]. Almost all missed cases (17 of 18, 94%) of INH resistance with genotypic testing were in INH mono-resistant cases, a finding found in a prior study [16]. Thus clinical consequences may be mitigated, as initial treatment regimens for INH mono-resistance incorporate standard first line therapy and outcomes of INH mono-resistance TB have been found to be similar to drug susceptible TB [21]. We found the most common genetic mutations conferring INH resistance were located in the *katG* gene at codon 531 with *inhA* mutations much less likely, consistent with previous reports [17], [18]. In our study, the MTBDR*plus* assay detected 97% of all RIF resistant cases with the most common genetic mutations occurring in the 530–533 base pair region of *rpoB* gene which also confirms two prior studies [7], [15].

The use of rapid and accurate tests for drug resistance detection offers hope in improving MDR-TB prevention and management through the early initiation of appropriate therapy. While no studies thus far have evaluated the clinical benefits of implementing rapid diagnostics, the potential benefits can be inferred from the drastic difference in time to results. Our study provides the most detailed data to date on the comparison of time to results in a real world setting and found that on average MDR-TB could be detected 17 or 67 days earlier with the MTBDR*plus* assay as compared to liquid and solid culture DST, respectively. When available in low and middle-income countries, solid media is more commonly used for AFB culture because of lower costs. The impact of rapid detection of MDR-TB should be substantial given that otherwise patients would have received more than two months of an inappropriate treatment (i.e., first line regimen) that could lead to further amplification of drug resistance prior to detection of INH and/or RIF resistance [4], [22], [23]. Using existing baseline DST data, an empiric MDR-TB regimen could be chosen with a week of TB diagnosis thus helping prevent further community and nosocomial spread of drug-resistant TB and limiting disease progression. Specific mutations found by the MTBDR*plus* assay may help in empiric choice of an anti-TB treatment regimen. If mutations are detected in only the *InhA* gene the isolate likely has low-level resistance to INH, and thus high dose INH may have clinical effect. Additionally, ethionamide inhibits *InhA* [24], and as our results demonstrate, if *InhA* mutations are present ethionamide resistance is highly likely. In contrast, while *katG* mutations indicate high-level INH resistance [20] so that INH would not be clinically effective, ethionamide could still be included in this scenario pending DST results.

Limitations of the MTBDR*plus* assay include detection of resistance to only RIF and INH, and the need for high-level technical skill and infrastructure usually relegating its use to a referral or regional laboratory. However, we were able to bring this technology to patients throughout the country of Georgia by referring sputum specimens that were AFB smear positive at local smear microscopy laboratories to the NRL. Continued surveillance through traditional culture and DST methods will remain important to individualize treatment regimens for drug-resistant TB and in the detection of XDR-TB. To aid in rapid diagnosis of XDR-TB, the Genotype MTBDR*sl* was recently developed to detect resistance mutations to fluoroquinolones, aminoglycosides, and ethambutol [25]. When used in combination with the MTBDR*plus* assay, it may allow the detection of XDR-TB within a week of TB diagnosis and could be triaged to be performed only in cases when there was resistance to RIF or both INH and RIF.

The study was subject to a few limitations including only enrolling patients with AFB smear-positive sputum specimens, not having information on HIV status, and no methods in place for identification of non-tuberculous mycobacterium (NTM). Based on available evidence, the WHO currently recommends line probe assays only in persons with an AFB smear-positive sputum [8]. Rates of HIV-TB co-infection in our cohort were likely low based on a prior study, which found an HIV prevalence of 1.1% [26] in tuberculosis patients in the Republic of Georgia. With no protocol for NTM identification, we were unable to confirm the presence of NTM in the six culture positive cases with a valid MTBDR*plus* assay result but no binding to the MTBC probe.

In conclusion, we have demonstrated that the MTBDR*plus* performed well in a “real world” situation at a NRL in a low and middle-income country with a high-burdened TB including MDR-TB. The line probe assay provided a much more rapid diagnosis of drug resistant TB including MDR-TB compared to convention laboratory tests (culture and DST). The line probe assay and other molecular diagnostic tests have the potential to significantly improve MDR-TB treatment, management and prevention by providing rapid diagnosis and helping to ensure patients are started on appropriate treatment regimens which will not amplify resistance. Ongoing studies, including an evaluation of this study cohort, are needed to help determine the impact on patient and program outcomes and optimal use of rapid TB diagnostic tests.
